# Hospital rating websites play a minor role for uro-oncologic patients when choosing a hospital for major surgery: results of the German multicenter NAVIGATOR-study

**DOI:** 10.1007/s00345-022-04271-1

**Published:** 2023-01-12

**Authors:** Christer Groeben, Katharina Boehm, Rainer Koch, Ulrich Sonntag, Tim Nestler, Julian Struck, Matthias Heck, Martin Baunacke, Annemarie Uhlig, Mara Koelker, Christian P. Meyer, Benedikt Becker, Johannes Salem, Johannes Huber, Marianne Leitsmann

**Affiliations:** 1 Department of Urology, Philipps-University Marburg, Baldingerstr., 35043 Marburg, Germany; 2 Department of Urology, University Hospital Mainz, Mainz, Germany; 3 Department of Urology, University Hospital of the Technical University of Dresden, Dresden, Germany; 4 Department of Urology, University Hospital Augsburg, Augsburg, Germany; 5 Department of Urology, Bundeswehrkrankenhaus Koblenz, Koblenz, Germany; 6 Department of Urology, University Hospital Brandenburg, Brandenburg Medical School Theodor Fontane, Brandenburg an der Havel, Brandenburg, Germany; 7 Department of Urology, University Hospital Luebeck, Luebeck, Germany; 8 Department of Urology, Klinikum Rechts der Isar der Technical University of Munich, Munich, Germany; 9 Department of Urology, University Medical Center Goettingen, Goettingen, Germany; 10 Department of Urology, University Medical Center Hamburg-Eppendorf, Hamburg, Germany; 11 Department of Urology, University Hospital Bochum, Ruhr-University Bochum, Campus OWL, Herford, Germany; 12 Department of Urology, Asklepios Hospital Barmbek, Hamburg, Germany; 13 Department of Urology, Klinik LINKS VOM RHEIN, Cologne, Germany; 14 Department of Urology, Medizinische Universitaet Graz, Graz, Austria

**Keywords:** Uro-oncologic surgery, Hospital rating websites, Survey, Decision making, Health services research

## Abstract

**Purpose:**

Hospital rating websites (HRW) offer decision support in hospital choice for patients. To investigate the impact of HRWs of uro-oncological patients undergoing elective surgery in Germany.

**Methods:**

From 01/2020 to 04/2021, patients admitted for radical prostatectomy, radical cystectomy, or renal tumor surgery received a questionnaire on decision-making in hospital choice and the use of HRWs at 10 German urologic clinics.

**Results:**

Our study includes *n* = 812 completed questionnaires (response rate 81.2%). The mean age was 65.2 ± 10.2 years; 16.5% were women. Patients were scheduled for prostatectomy in 49.1%, renal tumor surgery in 20.3%, and cystectomy in 13.5% (other 17.1%). Following sources of information influenced the decision process of hospital choice: urologists’ recommendation (52.6%), previous experience in the hospital (20.3%), recommendations from social environment (17.6%), the hospital's website (10.8%) and 8.2% used other sources. Only 4.3% (*n* = 35) used a HRW for decision making. However, 29% changed their hospital choice due to the information provided HRW. The most frequently used platforms were Weisse-Liste.de (32%), the AOK-Krankenhausnavigator (13%) and Qualitaetskliniken.de (8%). On average, patients rated positively concerning satisfaction with the respective HRW on the Acceptability E-Scale (mean values of the individual items: 1.8–2.1).

**Conclusion:**

In Germany, HRWs play a minor role for uro-oncologic patients undergoing elective surgery. Instead, personal consultation of the treating urologist seems to be far more important. Although patients predominantly rated the provided information of the HRW as positive, only a quarter of users changed the initial choice of hospital.

**Supplementary Information:**

The online version contains supplementary material available at 10.1007/s00345-022-04271-1.

## Introduction

Recent literature revealed relevant variability in the quality of care across health care providers in Germany as well as in the USA for uro-oncologic surgery [1–3]. At the same time, along with rising patient empowerment, quality of treatment is an important factor for patients when choosing a hospital [4]. Within this context, hospitals face growing requirements to release performance data to the public and therefore, potentially offer patients the opportunity to make granular-informed health care choices.

In Germany, various predominantly publicly financed initiatives provide public reporting of treatment-related quality information on specialized online platforms, so-called hospital rating websites (HRW) [5, 6]. These online platforms offer a comparison of hospital capacity, equipment and therapeutic options. Several offer service rating by the patient while one even displays treatment-specific quality scores using mortality and complication rates from routinely collected insurance data [5].

One fundamental idea of online public reporting through HRWs is to increase the overall quality of care by health care providers since it reveals differences in quality standards. This information gives patients the opportunity to choose better performing providers, thus motivating hospitals or departments to improve their respective outcomes [7, 8]. Previous systematic research has shown that public reporting has the potential to stimulate quality improvement outcomes at the provider level [9]. Especially the caseload of extensive uro-oncologic surgery poses a relevant economical aspect for healthcare providers. The most frequent and thus relevant interventions are radical prostatectomy, radical cystectomy and partial/radical nephrectomy [2, 10]. In Germany, a majority of uro-oncologic patients chooses their hospital by themselves [11]. However, motivations of patients for hospital choice are not sufficiently understood and evidence that patients make this choice based on performance data does not exist. Aim of the study is to evaluate the role of German HRW on patients undergoing major uro-oncologic surgery.

## Methods

Between 01/2020 and 04/2021, we performed a prospective multicentric cross-sectional survey-study at 10 German uro-oncologic centers. The hospitals were selected through the affiliation of the members of project-group “health services research” of the German-Society of Residents in Urology (GeSRU)-Academics. Since HRWs are called “Hospital-Navigators” in Germany, the study was named “NAVIGATOR.” Patients scheduled for either prostatectomy, cystectomy or (partial) nephrectomy due to prostate, urothelial or renal cancer were invited to take part in a printed survey the day of hospital admission. Patients younger than 18 years and patients with any other than the above-mentioned surgeries were not addressed. A written information about the study was handed out in advance. The survey was completely anonymized. The study was approved by the local ethics committee of the Medical University Goettingen (15/7/19), serving as waiver for the other participating centers.

The questionnaire contained 45 questions, in 4 categories. 20 questions had to be answered by all respondents, while 22 questions exclusively addressed users of a HRW and 3 questions the non-users. First, the participants answered 13 questions on their oncologic disease, stress, gender, age and socioeconomic characteristics. Then, 7 questions regarding media-usage, online-activity, decision-making and information-sources for hospital choice. Decisional preference was assessed using the Control Preference Scale (CPS) [12] and conflict with the Decisional Conflict Scale (DCS) [13]. Patients were asked whether they had used HRWs to choose the current hospital. This resulted in the HRW usage rate as one focus point of this study. This question led to a subdivision of patients into HRW-users and non-users. Twenty-two questions focused on the user experience with a HRW including a modified version of the Acceptability E-Scale for user-satisfaction with the website [14] and a question regarding the impact on their hospital choice. Three knowledge questions assessed the remembered content from the HRW. To evaluate the impact of the HRW use on the choice of hospital, HRW users were asked whether the HRW use had changed their previous decision. Non-users skipped this part and completed the questionnaire with three questions about the reasons against the use of a HRW and whether they would be willing to use one for future treatment decisions.

### Statistics

5% (*n* = 3) of the participants of a pilot-survey with 60 participants had used a HRW. We consequently hypothesized a HRW-usage of less than 10% in the target patient collective. The required number of cases to achieve statistical significance at a confidence interval (CI) width of 2.1% (7.9–12.1%) was calculated with 800 cases. Univariate analysis of significance was performed using Chi-square tests for categorical variables or t-tests for nominal variables. The difference of Incidence-Ratios was compared using the exact Poisson method. To demonstrate statistical correlation with age, age-groups were constructed (< 50, 50–59, 60–69, 70–79 and > 79 years of age). For multivariate analysis, linear and logistic models were implemented. HRW-usage and mental stress (Distress Thermometer) served as dependent variables in multivariate models to calculate the influence of the patient characteristics and prior information seeking on their mental stress. Since several questionnaires were not fully completed, extra groups of “missings” were excluded from testing for statistical significance. *p* < 0.05 was regarded as significant. For statistical analysis, SAS 9.4 (SAS Institute Inc., Cary, NC) was used.

## Results

### Baseline characteristics

Of 1000 distributed questionnaires, 829 were recollected. Seventeen questionnaires with missing answer to question 1 (“What disease do you suffer from?”) or question 20 (“Have you chosen the hospital with the help of a hospital navigator?”) were excluded from analysis, leaving 812 for evaluation (response rate 81.2%). A total of 134 of 812 respondents were female (16.5%). The overall mean age was 65.24 [standard deviation (SD) 10.17] years. Most received radical prostatectomy (49.0%), followed by surgery for renal tumors (20.2%) and radical cystectomy (13.5%), while 17.3% reported other indications. Table 1 gives an overview of the study population.

**Table 1:** Overview of the study sample (*n* = 812)

Characteristics	Study sample, *n* (%)
Age (years)	65.2 (SD 10.2)
Age categories (years)	
0–49	45 (5.5%)
50–59	140 (17.2%)
60–69	268 (33.0%)
70–79	213 (26.2%)
> 79	37 (4.6%)
Missing	109 (13.5%)
Gender	
Female	134 (16.5%)
Male	678 (83.5%)
Diverse	0
Disease	
Prostate cancer	398 (49.0%)
Bladder cancer	110 (13.5%)
Kidney cancer	164 (20.2%)
Other	140 (17.3%)
Previous urologic inpatient treatment	
Current clinic	205 (25.2%)
Different clinic	132 (16.2%)
None	364 (44.8%)
Not sure	3 (0.4%)
Missing	108 (13.4%)
Marital status	
Married	545 (67.1%)
Single	46 (5.7%)
Divorced	55 (6.8%)
Widowed	45 (5.5%)
Civil partnership	21 (2.6%)
Living separately	3 (0.4%)
Missing	96 (11.9%)
Monthly income	
< €1500	73 (9.0%)
€1500—€4000	270 (33.3%)
> €4000	114 (14.0%)
Missing	353 (43.5)
Insurance status	
Statutory	482 (59.4%)
Private	143 (17.6%)
Other	11 (1.4%)
Missing	176 (21.6%)
Residence (inhabitants)	
< 5000	205 (25.2%)
5000–20,000	153 (18.8%)
20,000–100,000	92 (11.3%)
100,000–1,000,000	91 (11.2%)
> 1,000,000	99 (12.2%)
Missing	172 (21.3%)
Graduation	
None	14 (1.7%)
Secondary school (Hauptschule/Mittlere Reife)	307 (37.8%)
Baccalaureate (Abitur)	75 (9.2%)
Technical college	75 (9.2%)
Polytechnic secondary school	46 (5.7%)
University degree	153 (18.8%)
Other	8 (1.0%)
Missing	134 (16.5%)
Language skills	
Native speaker	505 (62.2%)
Fluently	92 (11.3%)
Basic skills	15 (1.8%)
Missing	200 (24.6%)

### Hospital rating website use

**Fig. 1:**
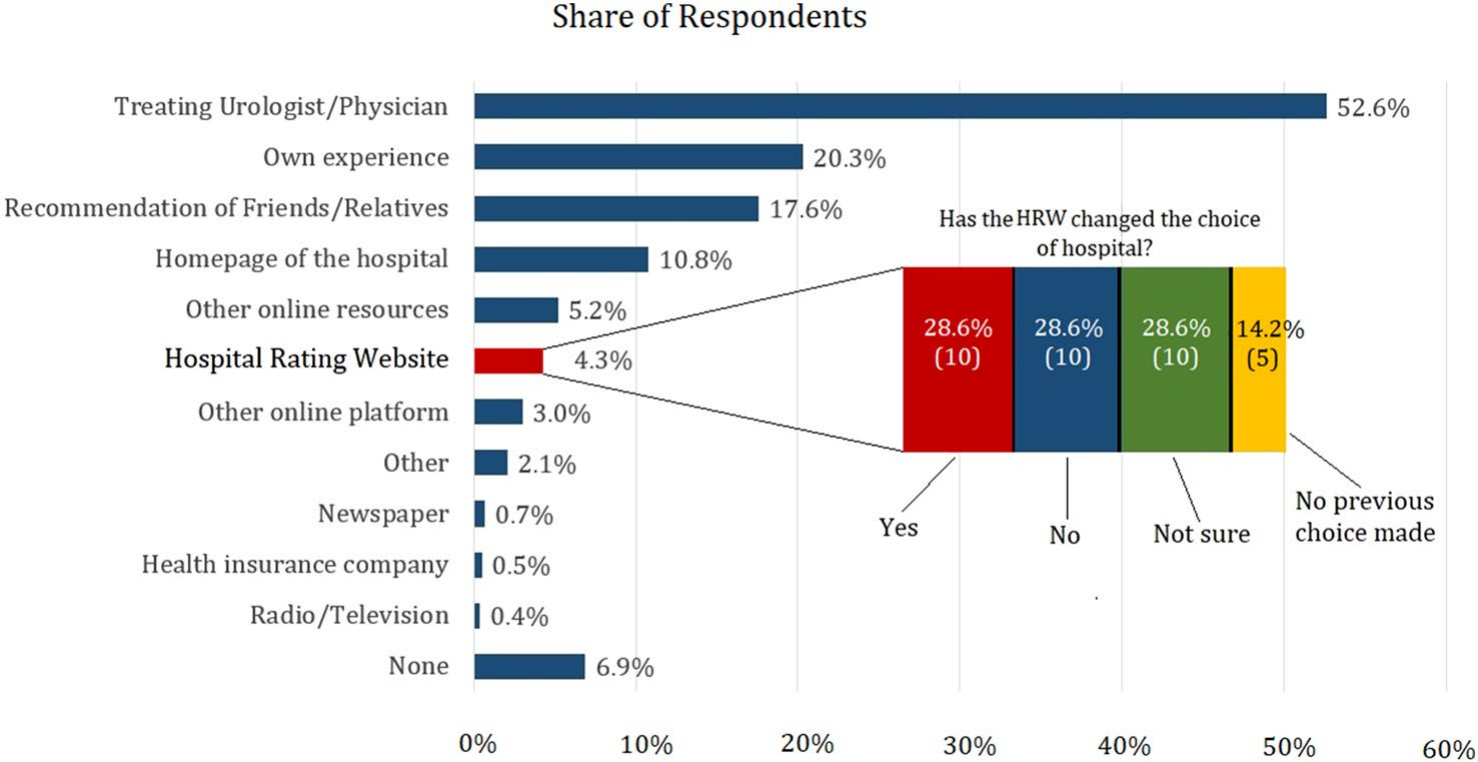
Sources of information for hospital choice (*n* = 812) and impact of the use of a hospital rating website (HRW) (*n* = 35)

Only 4.3% (*n* = 35) used a HRW for decision making, which confirmed the expected utilization rate of less than 10%. More than half (52.6%) stated that they had relied on the treating urologist as a source of information. Others reported personal experience with the hospital (20.3%), recommendations from relatives or friends (17.6%), and the clinic's homepage (10.8%) (Fig. 1). Of the HRW-users, however, 10 (28.6% of users, 1.2% of all participants) claimed to have changed their previous choice of hospital through use of a HRW. Non-users of an HRW predominantly claimed not having known about the possibility (47.0%; *n* = 365), followed by technical problems or lack of internet access (6.2%; *n* = 48) and distrust into HRW-providers (4.6%; *n* = 36) or the content (3.7%; *n* = 29). When asked if they would consider using a HRW in the future, 7.6% (*n* = 59) answered “yes,” while 19.6% (*n* = 153) responded “no,” 29.1% were unsure and 43.6% gave no answer.

Table 2 displays the different characteristics of the HRW-users and non-users. Patients with higher income and education more often used a HRW. HRW-users further showed higher internet use (daily use of 65.7%) and more often preferred a patient-centered decision making in the CPS [1.62 (SD 0.74)] compared to non-users [2.64 (SD 1.30)] (*p* < 0.01). When stratifying for surgical intervention 6.0% of the prostatectomy patients, 1.8% of cystectomy patients and 3.0% of kidney surgery patients (other 2.9%) reported having used a HRW. This showed no statistical significance (*p* = 0.12).

**Table 2:** Characteristics of users and non-users of a hospital rating website (HRW) (bold = significant differences)

Characteristics	HRW-users (*n* = 35)	Non-users (*n* = 777)	*p*
Age (years)	64.9 (SD 8.5)	65.3 (SD 10.2)	0.87
Gender			
Female	7 (20.0%)	127 (16.3%)	0.57
Male	28 (80.0%)	650 (83.5%)	
Diverse	0	0	
Disease			
Prostate cancer	24 (68.6%)	374 (48.1%)	0.12
Bladder cancer	2 (5.7%)	108 (13.9%)	
Kidney cancer	5 (14.3%)	159 (20.5%)	
Other	4 (11.4%)	136 (17.5%)	
Previous urologic inpatient treatment			
Current clinic	6 (17.1%)	199 (25.6%)	0.53
Different clinic	7 (20.0%)	107 (13.8%)	
None	18 (51.4%)	346 (44.5%)	
Not sure	0	3 (0.4%)	
Missing	4 (11.4%)	122 (15.7%)	
Monthly income			
< €1500	2 (5.7%)	71 (9.1%)	0.53
€1500—€4000	16 (45.7%)	254 (32.7%)	
€4000	7 (20.0%)	107 (13.9%)	
Missing	10 (28.6%)	345 (44.3%)	
Insurance status			
Statutory	24 (68.6%)	458 (58.9%)	0.25
Private	3 (8.6%)	140 (18.0%)	
Other	0	11 (1.4%)	
Missing	8 (22.8%)	168 (21.7%)	
Graduation			
None	1 (2.9%)	13 (1.7%)	0.75
Secondary school (Hauptschule/Mittlere Reife)	11 (31.4%)	296 (38.1%)	
Baccalaureate (Abitur)	2 (5.7%)	73 (9.4%)	
Technical college	4 (11.4%)	70 (9.0%)	
Polytechnic secondary school	2 (5.7%)	44 (5.7%)	
University degree	9 (25.7%)	144 (18.5%)	
Other	1 (2.9%)	7 (0.9%)	
Missing	5 (14.3%)	130 (16.7%)	
Regular media use			
Newspaper	19 (54.3%)	459 (59.1%)	0.74
Television	24 (68.6%)	533 (68.6%)	0.97
Radio	17 (48.6%)	411 (52.9%)	0.76
Computer	23 (65.7%)	418 (53.8%)	0.35
Smartphone	14 (40.0%)	222 (28.6%)	0.23
Missing	0	7 (0.9%)	0.57
Frequency of internet use			
None	1 (2.9%)	102 (13.1%)	0.13
Monthly	2 (5.7%)	15 (1.9%)	
Weekly	5 (14.3%)	77 (9.9%)	
Daily	23 (65.7%)	446 (57.4%)	
Missing	4 (11.4%)	137 (17.6%)	
Information sources for choosing the treating hospital			
Hospital navigator	35 (100%)	0	N/A
Other online platform	2 (5.7%)	44 (5.7%)	0.91
Homepage of the hospital	10 (28.6%)	78 (10.0%)	**0.01**
Other online resources	8 (22.8%)	34 (4.4%)	** < 0.01**
Treating urologist/physician	25 (71.4%)	412 (53.0%)	0.16
Recommendation of friends/relatives	8 (22.8%)	135 (17.4%)	0.44
Own experience	5 (14.3%)	157 (20.2%)	0.47
Newspaper	0	6 (0.8%)	0.60
Television/radio	0	3 (0.4%)	0.71
Health insurance company	1 (2.9%)	3 (0.4%)	0.17
Other	3 (8.6%)	14 (1.8%)	**0.04**
None	0	56 (7.2%)	0.11
Distress thermometer	5.55 (SD 3.08)	6.05 (SD 2.91)	0.38
Decisional conflict (DCS)	4.0 (SD 1.26)	4.16 (SD 0.96)	0.90
Control preference (CPS)	1.62 (SD 0.74)	2.64 (SD 1.30)	** < 0.01**

On multivariate analysis, no significant correlation between the possible influencing factors and the use of a HRW could be established. A multivariate analysis for the perceived mental stress at hospital admission, however, showed significantly lower levels of stress for patients with bladder cancer compared to kidney and/or prostate cancer as well as for younger (< 49 years) compared to older patients across both sexes (**Table ****3**). On univariate analysis, bladder cancer patients were more likely female (29.1 vs. 15.5%, *p* < 0.001), were significantly older (68.2 (SD 10.8) vs. 64.78 (SD 10.0) years, *p* = 0.002), were more likely pretreated in the current center (*p* < 0.001), reported less internet use (*p* = 0.46) and showed higher confidence with their choice of hospital (*p* = 0.14).

**Table 3:** Multivariate model of the prognostic factors for mental stress at the time of hospital admission assessed with the distress thermometer (intercept value = expected value of the distress thermometer in the reference group; bold = significant factors)

Characteristics	Women (*n* = 134)	*p*	Men (*n* = 678)	*p*
Intercept value	3.2372	–	3.5429	–
Disease				
Prostate cancer	–	–	0 (reference)	** < 0.001**
Bladder cancer	0 (reference)	**0.033**	**− 1.8384**	
Kidney cancer	**1.6210**		− 0.3332	
Other	**2.1230**		− 0.4028	
Age group (years)				
0–49	No significant influence	0.694	0 (reference)	**0.014**
50–59			0.7734	
60–69			0.8998	
70–79			**1.6915**	
> 79			1.4700	
Missing			**1.4780**	
No Newspaper/Smartphone use	0 (reference)	–	No significant influence	–
Regular media use				
Newspaper	**− 1.3258**	**0.044**		0.569
Smartphone	**1.6214**	**0.021**		0.839

Following investigated factors showed no significant correlation with mental stress:

Gender, marital status, income, insurance, graduation, frequency of internet use, information sources for choosing the treating hospital, use of hospital rating website

Of approximately 20 available HRWs in German language, “weiße-liste.de” was the most frequently cited by respondents (31.6%; *n* = 11), followed by the “AOK-Krankenhausnavigator” (14.2%; *n* = 5) and “Qualitätskliniken.de,” “Klinikbewertungen.de,” “Barmer Krankenhaus-Navi” achieving 8.6% (*n* = 3) each. On average, patients rated positively concerning satisfaction with the respective HRW (mean values of the individual items: 1.8–2.1). Usefulness of the HRW for decision-making was rated as particularly valuable (mean 1.8). Likewise, when participants were asked whether they would recommend the HRW to a friend or relative, 54% (*n* = 19) responded “yes,” while only 6% (*n* = 2) answered “no,” leaving the rest “unsure” or without any answer. Investigating the retained content on HRWs, the rate of respondents, who could not give a correct answer to this question, was relatively high (60 and 69%).

## Discussion

The impact of HRWs on patient’s hospital choice has been much debated in the past. Yet, evidence is lacking for the German healthcare system. Recent literature has demonstrated that public awareness and impact of hospital or physician rating websites tend to increase [15, 16]. In our study, less than 5% of all respondents reported the use of a HRW to support hospital choice. Furthermore, only about 29% (*n* = 10) of these (1.2% of all participants) reported to have changed their initial choice of hospital through use of the HRW. This demonstrates that for this specific patient cohort of uro-oncologic patients HRWs currently play a minor role. In addition, 45% (365/812) of all respondents claimed to have been unaware of the existence of HRWs. A comparable survey study showed rising publicity and usage rates of 1/3 of respondents [15]. However, these findings were acquired from an exclusively online population with healthy and significantly younger adults than in our cohort, possibly overestimating the impact of physician rating websites among the total population. Another study from the USA indicated high levels of awareness (75%) with over half respondents having used a HRW for their hospital search [17]. Again, the cohort was composed of younger (mean 45 years) patients with a high proportion of female participants and online-only users. According to findings from cross-generational surveys young and female respondents, more frequently rely on online-based sources when seeking information on health topics. [18, 19]. In current literature, younger age in general seems to be an independent factor for online health-related information seeking [20]. According to our results, urologic cancer patients are usually older and comorbid patient rather influenced by their urologist, friends and relatives or own experience.

Previous survey-studies among young and predominantly female cohorts reported rates of up to 80% of respondents describing a distinct impact of HRW-usage on hospital choice [17, 21]. In our study, only 29% of HRW-users agreed that using a HRW had changed the previous hospital choice. Past investigations showed that patients have been slow to take advantage of comparative reports when making a health care provider choice [22]. They were oftentimes unaware of the relevance of the presented information, incapable of understanding or mistrusted the provided information [23–25]. Thus, the contemporary uro-oncologic patient may not be the ideal patient for an online-based decision support for hospital choice. Urological clinics should be aware that the younger generations, who are nowadays mostly online and technology-affine, are the uro-oncological patients of tomorrow and thus, online-based support services for decision-making processes such as HRWs will become increasingly relevant.

Robust evidence exists, showing a reduction in decisional conflict or psychic stress in patients facing medical interventions through shared decision-making beforehand [26, 27]. However, our results showed no difference between HRW-users and non-users concerning these outcomes. This could be due to the low number of HRW-users. Only patients admitted for cystectomy, reported lower psychological stress. However, cystectomy patients had more often already been to the clinic due to the prior transurethral resection presumably thus reducing the stress. Making a HRW-supported choice or receiving information through media use beforehand seems to be secondary.

A limitation of our results is the restriction to the cohort of surgical cancer patients. However, uro-oncological surgery is of high financial importance for providers in Western industrialized nations [10, 28] and surgical expertise is mainly defined by case volume of major uro-oncological procedures required for cancer center certification in Germany [29]. Additionally, although the utilized questionnaire contained several validated items such as the CPS, the DCS, Distress Thermometer and the Acceptability-E-Scale, it has not yet been validated in its composed form. Another limitation is the relatively high proportion of incomplete questionnaires. Thus, we categorized the questions according to indispensable (questions 1 and 20) and included the proportion of questionnaires with missing answers in the results. Finally, the low number of HRW-users led to mostly statistically insignificant differences impairing the significance in terms of characterizing the HRW user cohort. These findings, in turn, are important for evaluating the current social relevance of HRWs and can serve as a baseline for future studies.

In summary, by providing a large multicenter collective that actually reflects our uro-oncology patient population, our survey provides insight for the first time into our patients’ decision-making processes for hospital choice and the respective influence of HRWs. Our results show that, despite the growing influence of HRWs in younger generations, the average German urologic cancer patient rarely uses HRWs for choosing his treating hospital for surgery and continues to rely predominantly on traditional sources of information such as advice from their urologist. This seems to be due to the mean high age of this cohort, with lower use of online resources. It can be assumed that this will increasingly change in the coming years and decades as the current younger generations with high media usage grow older. Thus, German uro-oncology clinics should be aware of HRWs as important source of information for their future patients. Understanding the processes and influential factors in decision making for hospital choice in different patient cohorts will be an existential issue for urology clinics in the near future. This could be supported by computerized process-tracing tools which allow analysis and prediction of decision-making strategies of individuals and whole population groups [30].

## Supplementary Information

Below is the link to the electronic supplementary material.

Supplementary file1 (DOCX 96 KB)

## Data Availability

The data that support the findings of this study are available from the corresponding author upon reasonable request.

## Abbreviations

HRW: Hospital rating website
CPS: Control Preference Scale
DCS: Decisional Conflict Scale
CI: Confidence interval
SD: Standard deviation
