# Supplementary material for: Hospital rating websites play a minor role for uro-oncologic patients when choosing a hospital for major surgery: results of the German multicenter NAVIGATOR-study
Source: World J Urol. 2023 Jan 12;41(2):601–9. doi: 10.1007/s00345-022-04271-1 (PMC9947074; doi:10.1007/s00345-022-04271-1)
Supplement: Supplementary file 1 — Supplementary file1 (DOCX 96 KB) [file 345_2022_4271_MOESM1_ESM.docx]

**Anonyme Befragung zur**

**Verwendung von Krankenhausnavigatoren in der Urologie (NAVIGATOR)**

Ihre Meinung ist uns wichtig!

*Sehr geehrte Patientin, sehr geehrter Patient,*

unsere Umfrage richtet sich an Patienten mit geplanter Operation bei Prostatakrebs, Harnblasenkrebs oder Nierentumoren. **Gerne möchten wir besser verstehen, wie Sie Ihren Weg in unsere Klinik gefunden haben und welche Rolle dabei sogenannte „Krankenhausnavigatoren“ gespielt haben.** Diese Internetseiten bewerten und vergleichen Krankenhäuser nach bestimmten Kriterien. Bekannte Beispiele hierfür sind die „Weiße-Liste“ oder der „AOK-Krankenhaus-Navigator“.

Die Teil­nah­me ist absolut freiwillig und eine Ablehnung ist für Sie mit keinerlei Nachteilen verbunden. Aus der Teil­nahme an dieser Umfrage ergibt sich für Sie persönlich kein direkter Nutzen. Gewonnene Erkenntnisse können jedoch die ärztliche Beratung verbessern und damit indirekt anderen Betroffenen nützen.

Ihre Angaben werden anonym erhoben und ausgewertet. Rückschlüsse auf einzelne Personen sind somit anhand der Daten nicht möglich. Ihre Angaben werden von uns vertraulich behandelt und nicht an Dritte weitergegeben. Die Ver­öffent­lichung der Studienergebnisse erfolgt ohne Bezug zu Ihrer Person.

**Bitte unterstützen Sie unsere Untersuchung mit der Beantwortung der folgenden Fragen (Die Bearbeitungszeit für alle Fragen beträgt etwa 10-15 Minuten):**

1. **An welcher Erkrankung leiden Sie?**

□ Prostatakrebs □ Harnblasenkrebs □ Nierentumor

□ Andere: _____________________________

1. **Bitte tragen Sie die Zahl ein (0 - 10), die beschreibt, wie belastet Sie sich in der letzten Woche einschließlich heute gefühlt haben.**

| 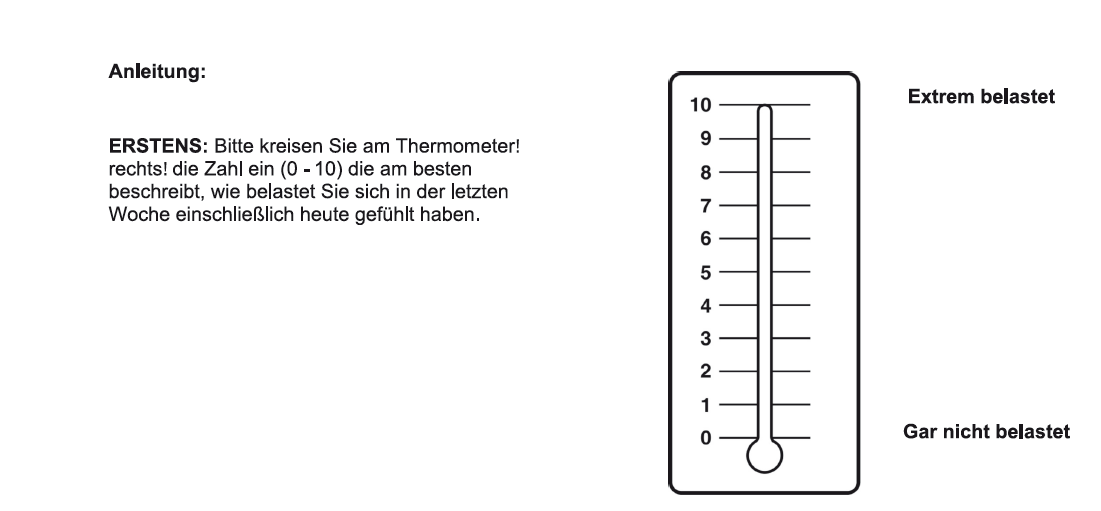 | **Tragen Sie bitte hier den Zahlenwert ein (1-10):**  **□** |
| --- | --- |

1. **Wurden Sie bereits an einer urologischen Klinik behandelt (Mehrfachauswahl möglich)?**

□ Ja, an dieser Klinik

□ Ja, an einer anderen Klinik

□ Nein

□ Weiß ich nicht

1. **Welches Geschlecht haben Sie?** □ weiblich □ männlich
2. **Wie alt sind Sie?** ______________ Jahre
3. **Wie ist ihr Familienstand?**

**□** Alleinstehend (z.B. auch geschieden, verwitwet)

□ Verheiratet oder in fester Partnerschaft

□ Sonstiges

1. **Wie hoch ist das monatliche Netto-Einkommen Ihres Haushaltes (Einkommen aller Haushaltsmitglieder abzüglich Steuern und Abgaben)? Bitte schätzen Sie den Betrag:**

□ < € 1.500 □ € 1.500 - € 4.000 □ > € 4.000 □ Keine Angabe

1. **Wie groß ist Ihr Wohnort (Einwohner):**

□ < 5.000 □ 5.000-20.000 □ 20.000-100.000 □100.000-1 Mio.

□ >1 Mio.

1. **Wie sind Sie krankenversichert?**

□ Gesetzlich □ Privat □ Sonstige

1. **Welchen Schulabschluss haben Sie?**

□ Keinen Abschluss

□ Hauptschulabschluss

□ Realschulabschluss

□ Polytechnische Oberschule

□ (Fach-) Abitur

□ Sonstiges

1. **Wie gut beherrschen Sie die deutsche Sprache?**

□ Muttersprache □ Fließend □ Grundkenntnisse

1. **Welche Medien nutzen Sie regelmäßig (Mehrfachnennungen möglich)?**

**□** Zeitung/Zeitschrift □ Fernsehen □ Radio □ Computer/Internet

□ Mobile App (Smartphone/Tablet) □ Andere

1. **Wie häufig nutzen Sie das Internet (über Computer/ Smartphone/ Tablet usw.):**

□ keine Nutzung □ monatlich □ wöchentlich □ täglich

1. **Wer hat die Entscheidung getroffen, in welcher Klinik Sie sich behandeln lassen? Diese Entscheidung habe ich...**

□ selbständig getroffen.

□ selbst getroffen, aber die Meinung meines Arztes einbezogen.

□ mit meinem Arzt gemeinsam getroffen.

□ meinem Arzt überlassen, der meine Meinung einbezogen hat.

□ meinem Arzt alleine überlassen.

1. **Sie haben sich für eine Behandlung an unserer Klinik entschieden. Wie sicher fühlen Sie sich mit dieser Entscheidung?**

□ sehr unsicher □ unsicher □ weder noch □ sicher □ sehr sicher

1. **Welche Informationsquellen haben Sie genutzt, um die für Sie passende Klinik zu finden (Mehrfachauswahl möglich)?**

□ Online-Krankenhausnavigator (Beispiele in Frage 18)

□ Andere Krankenhausbewertungen im Internet (z.B. Google-Rezension)

□ Internetauftritt dieser Klinik (Homepage)

□ Sonstige Internetquellen

□ Arztgespräch

□ Empfehlung von Freunden/Verwandten

□ Persönliche Erfahrung mit dieser Klinik

□ Zeitung/Zeitschrift (z.B. Focus-Liste)

□ Fernsehen/Radio

□ Beratungsangebot Ihrer Krankenkasse (z.B. Telefonberatung)

□ Andere: ____________________________________________

□ Keine

1. **Haben Sie sich für die behandelnde Klinik mithilfe eines Krankenhausnavigators entschieden? (Beispiele Frage 18)**

□ Ja => Bitte fahren Sie mit Frage 18 auf der nächsten Seite (Seite 4) fort.

□ Nein => Bitte fahren Sie mit Frage 37 (Seite 7) fort.

1. **Welche Krankenhausnavigatoren oder Bewertungsportale haben Sie genutzt (Mehrfachauswahl möglich)?**

□ Weiße Liste.de □ Sanego.de

□ AOK-Krankenhausnavigator □ DAK-Klinikführer

□ Klinikbewertungen.de □ Krankenhaus.de

□ TK-Klinikführer □ BKK Klinikfinder

□ Barmer Krankenhausnavi □ klinikfinder.kkh.de

□ Jameda.de □ HEK-Klinikfinder

□ Regionale Krankenhausspiegel □ IKK-Kliniksuche

□ Qualitätskliniken.de

□ Deutsches Krankenhausverzeichnis.de

□ Andere _______________________________________________

*Sollten Sie mehrere Krankenhausnavigatoren genutzt haben, gehen Sie bitte im Folgenden von jenem aus, der Ihre Entscheidung am stärksten beeinflusst hat und markieren Sie diesen in der vorhergehenden Frage (Frage 18) deutlich, indem Sie den Namen einkreisen.*

1. **Wie sind Sie auf den Krankenhausnavigator aufmerksam geworden?**

□ Internet

□ Arztgespräch

□ Empfehlung von Freunden/Verwandten

□ Zeitung/Zeitschrift

□ Fernsehen/Radio

□ Krankenkasse

□ Andere: ___________________

1. **Wie haben Sie den Krankenhausnavigator genutzt?**

□ alleine

□ Mit meiner Partnerin/meinem Partner

□ Mit Freunden/Verwandten

□ Mit anderen

1. **Haben Sie das bestbewertete Krankenhaus ausgewählt?** □ Ja □ Nein □ Weiß ich nicht
2. **Hat der Krankenhausnavigator Ihnen diese Klinik empfohlen?**

□ Ja □ Nein □ Weiß ich nicht

1. **Welche Information aus dem Krankenhausnavigator war für Ihre Entscheidung ausschlaggebend?**

___________________________________________________________

1. **Enthielt der Krankenhausnavigator eine Rangliste mit dem Vergleich von Eigenschaften mehrerer Krankenhäuser?** □ Ja □ Nein □ Weiß ich nicht
2. **Verwendete der Krankenhausnavigator Bewertungen von ehemaligen Patienten (z.B. mittels Schulnoten)?** □ Ja □Nein □ Weiß ich nicht
3. **Verwendete der Krankenhausnavigator Kommentare von ehemaligen Patienten?**

□ Ja □ Nein □ Weiß ich nicht

1. **Welcher Anbieter betreibt den genutzten Krankenhausnavigator?**

________________________________ □ Weiß ich nicht

1. **Wie einfach konnten Sie den Krankenhausnavigator nutzen?**

□ sehr einfach □ einfach □ mittel □ schwer □ sehr schwer

1. **War der Inhalt für Sie verständlich?**

□ sehr einfach □ einfach □ mittel □ schwer □ sehr schwer

1. **Wie hat Ihnen die Nutzung des Krankenhausnavigators gefallen?**

□ sehr gut □ gut □ mittel □ schlecht □ sehr schlecht

1. **Wie hilfreich war der Krankenhausnavigator dabei eine Klinik auszuwählen?**

□ sehr hilfreich □ hilfreich □ mittel □ wenig hilfreich □ nicht hilfreich

1. **War der Zeitaufwand zur Nutzung des Krankenhausnavigators angemessen?**

□ sehr angemessen □ angemessen □ mittel □ unangemessen □ sehr unangemessen

1. **Wie würden Sie insgesamt Ihre Zufriedenheit mit dem Krankenhausnavigator beschreiben?**

□ sehr zufrieden □ zufrieden □ mittel □ unzufrieden □ sehr unzufrieden

1. **Würden Sie den Krankenhausnavigator einem anderen Patienten weiterempfehlen?**

□ Ja □ Nein □ Weiß ich nicht

1. **Welche Informationen aus dem Krankenhausnavigator haben Ihnen am meisten geholfen? Bitte wählen Sie bis zu 3 Antworten!**

□ Rangliste

□ Bewertung durch andere Patienten

□ Technische Ausstattung

□ Entfernung zu Ihrem Wohnort

□ Erfahrungsberichte anderer Patienten

□ Jährliche Fallzahl Ihrer Operation

□ Patientensicherheit

□ Hygiene

□ Zertifizierung/Qualitätsmanagement

□ Sterblichkeitsrate

□ Komplikationsrate

□ Komfort (Zimmer, Essen, Fernsehen)

□ Organisation (z.B. Wartezeit)

□ Andere _______________________________________________

1. **Hat sich durch die Nutzung des Krankenhausnavigators ihre ursprüngliche Klinikwahl verändert?**

□ Ja □ Nein □ Weiß ich nicht

□ Ich hatte zuvor noch keine Klinik ausgewählt

***Wenn Sie einen Krankenhausnavigator genutzt haben ist hier der Fragebogen beendet.***

***Vielen Dank für Ihre Unterstützung!***

***Haben Sie noch keinen Krankenhausnavigator benutzt? Dann geht es hier weiter:***

1. **Was waren Ihre Gründe gegen die Nutzung eines Krankenhausnavigators (Mehrfachauswahl möglich)?**

□ Technische Probleme □ Kein Internetzugang

□ Zeitaufwand □ Misstrauen gegenüber Inhalten

□ Misstrauen gegenüber Anbietern □ Möglichkeit war nicht bekannt

□ Schlechte Erfahrungen in der Vergangenheit

□ Andere ____________________________

1. **Würden Sie zukünftig einen Krankenhausnavigator nutzen?**

□ Ja □ Nein □ Vielleicht

***Der Fragebogen ist hiermit beendet.***

***Vielen Dank für Ihre Unterstützung!***
